# Ways of Coping with Stress among Patients with Depressive Disorders

**DOI:** 10.3390/jcm11216500

**Published:** 2022-11-02

**Authors:** Agata Orzechowska, Katarzyna Bliźniewska-Kowalska, Piotr Gałecki, Agata Szulc, Olga Płaza, Kuan-Pin Su, Dan Georgescu, Małgorzata Gałecka

**Affiliations:** 1 Department of Adult Psychiatry, Medical University of Lodz, 91-229 Lodz, Poland; 2 Department Psychiatry, Faculty of Health Sciences, Medical University of Warsaw, 05-802 Pruszków, Poland; 3 Department of Psychiatry & Mind-Body Interface Laboratory (MBI-Lab), China Medical University Hospital, Taichung 404, Taiwan; 4 An-Nan Hospital, China Medical University, Tainan 709, Taiwan; 5 Department of Consultation-Liaison Psychiatry, Old Age Psychiatry and Neuropsychiatry, Psychiatric Services Aargau, 5210 Windisch, Switzerland; 6 Department of Psychotherapy, Medical University of Lodz, 91-229 Lodz, Poland

**Keywords:** stress, depression, coping with stress

## Abstract

Background: Experiencing stressful life events and ways of coping with them can predispose to the onset of depressive mood disorders, while depression itself can be responsible for severe stress and can weaken resilience to stressors. Thus, variables relevant to the onset of depressive episodes and the course of depression have significant relationships with coping strategies to stressors. The aim of this research was to evaluate the most commonly used stress-coping strategies in patients treated for depression compared to patients with anxiety disorders and to healthy subjects. Methods: The multidimensional coping inventory (COPE Inventory) by C. S. Carver, M. F. Scheier, and J. K. Weintraub, covering 15 stress response strategies included in more general and overarching coping styles, was used in the study. Results: Patients with depression differed from the healthy subjects in a statistically significant way. Statistical analysis showed that people with depression are less likely than healthy subjects to use *Active Coping, Planning, Seeking of Instrumental and Emotional Social Support, Suppression of Competing Activities, and Positive Reinterpretation*. In contrast, they are more likely to use *Denial, Mental Disengagement*, and *Behavioral Disengagement* compared to those not treated for mental disorders. The patients with depressive disorders, compared to the group of patients with anxiety disorders, scored significantly differently on stress coping strategies in only two types of actions taken in stressful situations. Conclusion: The patients with depression differed from the healthy subjects in terms of the highest number of the stress coping strategies assessed. Compared to the healthy individuals, a tendency toward an avoidant behavior style was prevalent among the depressed patients. There was no statistically significant difference between the patients with the first episode of the disease and recurrent depressive disorders in terms of stress coping strategies.

## 1. Introduction

According to the transactional theory of stress [1,2], which is one of the most popular theories on stress, coping with stress is a cognitive and behavioral effort directed at meeting specific external and/or internal demands that are judged to exhaust or exceed an individual’s resources. Thus, it is a process encompassing all of an individual’s efforts to cope with a given situation. Assessing the situation in terms of stress entails evaluating the possibility of taking action to eliminate the causes of stress or mitigate its effects. It represents an adaptive process, the course of which depends on the primary and secondary assessments made by the subject. In the concept by Richard Lazarus and Suzan Folkman [1,2], a person evaluates his or her capabilities, competencies, experienced social support or material resources, which can be useful for restoring balance between him or her and the environment. It is the secondary assessment that is the starting point for activity aimed at changing the stress transaction, which has been referred to as stress coping [3]. In addition, Lazarus and Folkman [1,2] distinguished two main functions of coping, namely instrumental-referring to problem-focused coping, and regulatory-related to emotion-focused coping strategies.

An individual’s health and mental condition depends on the nature of stressful events and the efforts made to transform them favorably. Thus, effective coping with stress is an important determinant of human health, contingent on both the outcome of coping and coping strategies (ways). The choice of strategy depends not only on the perception of the given situation—taking into account the context of the stressful event—or the coping style characteristic of the person, but also on personality dispositions [4,5].

Depression is one of the most common mental disorders, frequently associated with sadness, dejection, low self-esteem and decreased activity. In extreme states of severity, it may lead to discouragement about life, resigned attitudes and even suicidal intentions. This disease is simultaneously a syndrome of symptoms that includes both first time and recurrent depressive episodes.

Models explaining the etiology of depression primarily consider biological factors relating to the genetic background, dysregulation within neurotransmission and neuroanatomical dysfunction, the action of the pituitary-hypothalamic-adrenal axis, and the activity of pro-inflammatory cytokines. Much attention in shaping the determinants of depression is also given to psychological and environmental factors, which in their theoretical assumptions emphasize the importance of personality traits, a person’s early experiences and adaptation to environmental demands, including ways of coping with stress or how one cognitively elaborates one’s experiences and evaluates oneself. Contemporary research shows that a holistic approach, an attempt to bring together the reports described, drawn from both medicine and psychology or even sociology [6], seems most pertinent.

Stress is one of the elements that increases the risk of falling ill included in the multifactorial determinants of somatic and mental diseases and disorders. Frequently, the subject of research in the case of depression is the relationship of stress to its onset, the role of stress in inducing subsequent phases of the disease, and all those factors that mediate the stress-depression relationship [7,8]. In pathophysiological terms, depressive disorders resemble chronic stress. The inflammatory theory of depression portrays it as a chronic “cold” of the organism in response to stressors that occur during life [9]. Both physical and psychological (emotional) stress increases the likelihood of depressive disorders through the action of a number of hormonal, biochemical, and epigenetic mechanisms. These include: dysregulation of the hypothalamic-pituitary-adrenal axis under the influence of stressors, resulting in numerous hormonal changes; chronic inflammatory processes in the body; weakening of the immune system; and changes in the functioning of selected brain structures and in brain neurotransmission, which can cause, among other things, clinical symptoms of depression [7,8,9,10].

The relationship between the phenomenon of stress and depression is not unidirectional. Stress can directly affect an individual’s health by activating physiological mechanisms that occur in response to stressors, as well as, indirectly, through coping strategies undertaken in stressful situations. Experiencing stressful life events and ways of coping with them can predispose to the onset of depressive mood disorders, while depression itself can be responsible for severe stress and can weaken the techniques developed to resist it. Thus, variables relevant to the onset of depressive symptoms and the course of depression have significant relationships with coping strategies in the face of stress [8].

The fundamental purpose of the research presented herein was to evaluate the most commonly used coping strategies for dealing with stressful situations by people treated for depression compared to patients with anxiety disorders and in relation to healthy subjects. The reason for undertaking the described research, in comparison to the previous-preliminary results, was that the authors decided to increase the number of respondents and compare them to another disease entity.

## 2. Materials and Methods

A group of 190 people were invited to participate in the study, including 118 patients treated for mental disorders, i.e., depressive disorders: mild depressive episode (F32 according to ICD-10), recurrent depressive disorder (F33 according to ICD-10) [11], aged 18–66 years (M = 47.82; SD = 11.88), 75 women and 43 men. There were 33 patients (27.97%) with the first depressive episode (F32 according to ICD-10), while recurrent depressive disorder (F33 according to ICD-10) was diagnosed in 85 (72.04%) of the remaining patients. The duration of illness for those with recurrent depression was 1 to 2 years (23 people), 3–5 years (33 people), 6–10 years (12 people), and more than 10 years (17 people). The patients with the first depressive episode chose a duration interval of less than 1 year. 

The severity of depressive symptoms was measured on the first day of the patients’ hospitalization using the 17-item Hamilton Depression Scale (HAM-D) [12]. The mean score obtained by all the patients on the first day of the study was M = 22.24; SD = 6.36, corresponding to severe depression. The patients were being hospitalized when the analysis of the psychological test, the Hamilton test and a brief sociodemographic questionnaire were performed. 

**Figure 1:**
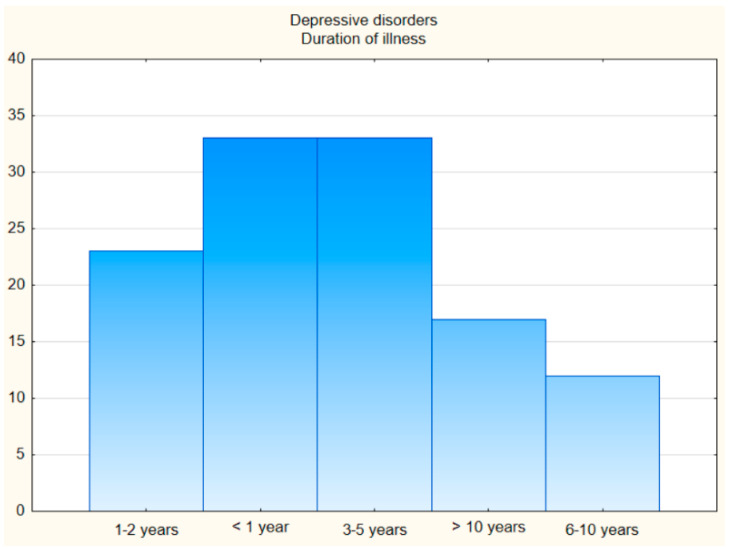
The duration of illness.

**Figure 2:**
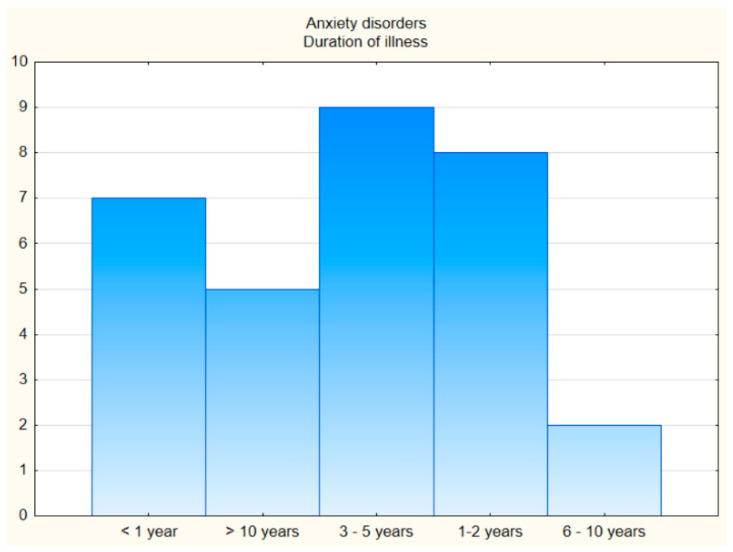
The duration of illness.

**The first comparison group** consisted of 31 patients with **anxiety disorders** in the form of phobic anxiety disorders (F40 according to ICD-10) and other anxiety disorders (F41 according to ICD-10), ranging in age from 19 to 60 (M = 36.06; SD = 11.50), 18 women and 13 men. The duration of illness in the group of patients with anxiety disorders was as follows: up to 1 year (7 people), 1–2 years (8 people), 3–5 years (9 people), 6–10 years (2 people), and more than 10 years (5 people) (Figure 1 and Figure 2).

**The second comparison group** was made up of 41 **healthy subjects**, including 26 women and 15 men between the ages of 20 and 61 (M = 32.95, SD = 11.61).

The most important data describing the individuals who were invited to the study are shown in Table 1.

The respondents completed the Stress Coping Questionnaire at the beginning of hospitalization. The COPE Inventory (multidimensional coping inventory) by C. S. Carver, M. F. Scheier, and J. K. Weintraub, in an adaptation by Z. Juczyński and Ogińska-Bulik, was used in the study. In 1989, Carver et al. [13] attempted to combine coping with stress understood as a style and as a strategy, referring to the transactional theory of stress and the self-regulation model of behavior. They proposed more than a dozen coping strategies which can reflect both a certain constant tendency and ways of dealing with a specific situation. To assess those strategies, they created the COPE (Coping Orientation to Problems Experienced) Inventory. This self-description-based tool consists of 60 statements that are answered on a 4-point scale: *1 = I usually don’t do this at all; 2 = I usually do this a little bit; 3 = I usually do this a medium amount; 4 = I usually do this a lot.* It allows 15 strategies for responding to stressful situations to be assessed. These strategies are:

1. Active Coping (taking action to remove, reduce the stressor or its effects)
2. Planning (considerations for dealing with stressors)
3. Seeking of Instrumental Social Support (seeking advice, help or information)
4. Seeking of Emotional Social Support (seeking moral support, sympathy or understanding)
5. Suppression of Competing Activities (avoiding other activities that are not related to the problem in order to cope better)
6. Turning to Religion (religion as a source of emotional support, a signpost for positive re-evaluation and development)
7. Positive Reinterpretation (seeing value and opportunity in an event for development, seeing it in a more positive light)
8. Restraint Coping (refraining from acting prematurely, waiting for the right moment)
9. Acceptance (accepting the situation as irreversible, something to get used to and learn to live with)
10. Focus on and Venting of Emotions (concern about one’s own emotions and the tendency to vent them)
11. Denial (ignoring, rejecting the fact of the situation)
12. Mental Disengagement (avoiding thinking about the consequences of an event by attending to other activities, e.g., sleeping, watching TV)
13. Behavioral Disengagement (helplessness, abandonment of efforts to achieve goals)
14. Substance Use (use of alcohol for temporary relief of unpleasant emotions)
15. Humor (considered as a way to alleviate unpleasant emotions)

The 15 stress response strategies described are part of more general and overarching coping styles, which include problem-focused, avoidant coping, support-seeking and emotion-focused styles. The strategies examined by the COPE Inventory reflect both a fixed tendency to cope in a certain way (dispositional coping) and coping in a specific stressful situation (referred to as situational coping).

The patients with depressive disorders and anxiety disorders were on antidepressant and anti-anxiety pharmacological treatment as prescribed by their attending physicians, and participation in the study was not associated with a modification in the ongoing pharmacological therapy. Individuals (both patients and healthy individuals) with other concomitant psychiatric diagnoses within Axis I and Axis II disorders were excluded from the study. Severe or chronic somatic diseases (neurological, autoimmunological, oncological) were additional exclusion criteria for all the study groups. Only individuals with negative history for psychiatric disorders were included. The study was conducted in accordance with the rules of the Data Protection Act, and its design was approved by the Bioethics Committee (RNN/882/11/KB). The subjects provided written informed consent to participate in the study. 

STATISTICA 13.3 PL software was used to perform a statistical analysis of the results obtained. During a statistical verification of the hypotheses, a two-tailed critical area was assumed. In order to choose the type of measurement, an analysis of the variables under study was conducted, which showed that the hypothesis of conformity to a normal distribution should be rejected. In order to demonstrate the statistical significance of the relationship between the analyzed variables, a statistical analysis was performed based on non-parametric tests, also in relation to group size. The Mann-Whitney U test as well as Spearman’s rank correlation were used. The significance level was *p* < 0.05 in all the statistical methods applied. 

## 3. Results

In order to organize the presentation of the statistical analysis results, letter numbering was used.

**(A)** Before making a comparative analysis between the groups, the results obtained by each group of respondents were analyzed. 

In the group of patients treated for **mood disorders** (first depressive episode and recurrent depressive disorder), the subjects scored highest consecutively (from the highest score) on scales describing the following strategies of coping with stressful situations:

- *Focus on and Venting of Emotions*
- *Active Coping*
- *Restraint Coping*
- *Planning*

Scores on these scales oscillated at values between 10 and 11 points (the possible highest score was 16, the lowest was 4 points).

In the questionnaire described above, the patients with **anxiety disorders** received the highest scores in the following strategies:

- *Focus on and Venting of Emotions*
- *Seeking of Instrumental Social Support*
- *Seeking of Emotional Social Support*
- *Active Coping*
- *Planning*

As among the patients with depression, scores on these scales oscillated at values between 10 and 12 points.

The respondents from the **healthy** group most often used the following strategies in difficult situations (in order of the highest score):

- *Planning*
- *Seeking of Instrumental Social Support*
- *Active Coping*
- *Positive Reinterpretation*
- *Focus on and Venting of Emotions*

The scores on these scales oscillated at values between 11 and 12 points. 

A review of the mean values in the scales indicated that the healthy subjects and patients with anxiety disorders were more likely to choose more favorable coping strategies compared to those with depression. 

**(B)** Statistical analysis of the differences between the mean values for the 15 strategies used in coping with stress, obtained by all those invited to the study, showed statistical significance for most of the variables studied. The patients with depression differed from the healthy subjects in a statistically significant way in terms of the highest number of the stress coping strategies assessed. Statistical analysis showed that people with depression are less likely than healthy subjects to use *Active Coping, Planning, Seeking of Instrumental and Emotional Social Support, Suppression of Competing Activities, and Positive Reinterpretation*. In contrast, they are more likely to use *Denial, Mental Disengagement*, and *Behavioral Disengagement* compared to those not treated for mental disorders. This means that the tendency for this group of patients to adopt an avoidant behavioral style is prevalent compared to healthy individuals. The results obtained are presented in Table 2.

The above analysis of differences in the use of specific stress coping strategies was also carried out with a breakdown of the type of diagnosis in terms of mood disorders for which the patients studied were being treated. There was no statistically significant difference between the patients with the first episode of the disease and recurrent depressive disorders in terms of stress coping strategies. The subjects differed only in the *Humor* strategy (Z = −2.13, *p* = 0.034). The described strategy is more often used by people with the first episode of the disease. In the group of patients with the first depressive episode compared to the healthy subjects, the patients differed from comparison group 2 in terms of the six strategies analyzed. In contrast, the patients with recurrent depressive disorder differed from the healthy individuals in as many as 10 coping strategies.

The patients with depressive disorders, compared to the group of patients with anxiety disorders, scored significantly differently on stress coping strategies in only two types of actions taken in stressful situations, namely *Seeking of Instrumental Social Support and Seeking of Emotional Social Support.* In both strategies, those with depression scored statistically significantly lower than those with anxiety disorders (Table 3).

To broaden the analysis of the stress-related psychological factors studied, a comparison was made between the patients with anxiety disorders and the healthy individuals; the patients with anxiety disorders differed from the healthy subjects in a statistically significant way with reference to *Active Coping, Planning, Suppression of Competing Activities, Positive Reinterpretation, Acceptance,* as well as *Humor*, scoring lower in these strategies than the healthy individuals. In the group of patients with anxiety disorders, similarly to among those with depression, the following strategies were used more often than among the healthy subjects: *Denial* and *Behavioral Disengagement.*

**(C)** To looking for comorbidity between the severity of depressive symptoms, as measured by the Hamilton Depression Scale (HAM-D) and the stress coping strategies used, a statistical analysis of Spearman’s rank correlation was performed. The results of the calculations showed that higher levels of mood disorder symptom intensity were statistically significantly associated with only one strategy used during stressful events, i.e., *Behavioral Disengagement* (R = 0.23; *p* = 0.016). The greater the severity of depression, the more frequent the use of this strategy. As the level of severity of depressive symptoms increases, a person’s typical coping strategies do not change. 

**(D)** For patients with depression, correlation of illness duration with individual coping strategies showed that longer illness duration statistically significantly co-occurred with three out of fifteen coping strategies, namely *Denial*, *Mental Disengagement*, and *Humor*. The longer the patients had been ill in the study population, the less inclined they were to use the given coping strategies. The results are presented in Table 4.

In the group of patients with anxiety disorders, in comparison to those with depression, illness duration did not correlate statistically significantly with stress coping strategies. 

**(E)** The gender of the subjects had little effect on the type of coping strategies chosen to deal with stressful situations. For the entire study population (190 individuals), women differed from men in the following strategies: *Seeking of Emotional Social Support* (Z = −3.02; *p* = 0.003), *Turning to Religion* (Z = −2.65; *p* = 0.008) and *Focus on and Venting of Emotions* (Z = −2.28; *p* = 0.023), in which the women obtained a higher score than the men. The patients with depressive disorders differed by gender only in terms of *Turning to Religion* (Z = −2.61; *p* = 0.009), which is more often used by female respondents in this group. In the anxiety disorder group, women did not differ significantly from men in any of the coping strategies used to deal with stress. For those without mental illness, women reported more frequent use of the following strategies: *Seeking of Emotional Social Support* (Z = −2.65; *p* = 0.008) and being less likely to *refrain from action* (Z = 2.11; *p* = 0.035).

**(F)** The subjects with depression differed statistically significantly in terms of mean age value from the individuals with anxiety disorders and from the healthy subjects, as shown in Table 1, which presents the characteristics of the subjects. This has to do with both the nature of the mental illness itself, in terms of the typical average age of onset for the disease, and the availability of the subjects during their hospitalization. At the same time, correlation of age with individual ways of coping with stress showed negative statistical significance in this group only in the *Mental Disengagement* strategy. In comparison, the statistical significance of this association among the patients with anxiety disorders was observed only for *Turning to Religion* (positive correlation) and *Humor* (negative correlation); among the healthy subjects it was only visible for *Focus on and Venting of Emotions* (positive correlation) and *Behavioral Disengagement* (positive correlation), while with reference to entire study population only for *Humor* (negative correlation).

## 4. Discussion

Affective diseases, including depression, are one of the leading causes of psychiatric hospitalization. At least 15% of adult women and 10% of men are at risk of developing the disease. The most prominent symptoms of affective diseases involve the emotional sphere, which significantly affects the functioning of patients. The determinants of depressive disorders include both biological variables, such as genetic predisposition, neuroanatomical or immunological factors, and psychosocial (environmental and personality) factors [14]. 

The variables relevant to the onset of depressive symptoms and the course of depression have significant relationships with coping strategies in the face of stress. Treating stressors as a threat and lacking constructive coping strategies and styles, along with neuroticism and high perfectionism, low levels of commitment to action, low self-esteem, lack of a sense of control over the situation, low social skills and a pessimistic attribution style, are predisposing factors for depression [15].

There have been many scientific reports as a result of studies of the relationship between the phenomenon of stress and depression. However, the literature has paid somewhat less attention to a thorough analysis of the coping strategies used to deal with stressful situations inthese patients. It is not always the case that the choice of stress coping strategies is explicitly linked to health. Past studies also include those that have found a negligible association between the frequency of using various stress coping strategies and subjective self-assessment of health, which may mean that what is more important for mental and physical well-being is not so much the number of strategies chosen but their adequacy [16]. 

This publication shows that people with depressive disorders (first episode and recurrent depressive disorders) differ statistically significantly from healthy individuals in terms of the stress coping strategies they use. Among people with depression, strategies categorized as avoidant behavior style are dominant compared to healthy people, i.e., *Denial, Mental Disengagement* and *Behavioral Disengagement*. Compared to those without depression, depressed patients use strategies that fit into the problem-focused style, which consists of *Active Coping, Planning, Suppression of Competing Activities* and *Positive Reinterpretation*, significantly less often. In contrast, the strategies of *Seeking of Instrumental Social Support* and *Seeking of Emotional Social Support* are just as rarely used among patients with mood disorders. In its course, depression is most often associated with passivity, withdrawal from action, a resigned attitude and lack of initiative. 

Similar results from a preliminary study of our own [17] showed that patients treated for depressive disorders in stressful situations are more likely than healthy individuals to use strategies based on problem avoidance and active coping as well as denial of the problem, and have more difficulty seeing the positive aspects of stressful events. In reports from 2013, men and women in the general population of people studied differed in the small number of ways they chose to cope with stressful situations. This is also confirmed by the current results of the analysis of the described variables. The results described suggest that depressive-type mood disorders may be a significant factor in the negative evaluation of one’s own coping skills and a greater tendency to view stressful events as a threat which is beyond one’s ability to cope [17]. 

Research results confirming the use of less effective ways of coping with stress in people with depressive symptoms were also demonstrated in a group of 2057 medical students from Chongqing Medical and Pharmaceutical College in China, who were surveyed using a self-report questionnaire that included demographic data, the Zung Self-Rating Depression Scale (SDS), the Zung Self-Rating Anxiety Scale (SAS), the Family APGAR Index, the Social Support Rating Scale, and the Coping Strategies Questionnaire (CSQ). Depression correlated negatively with positive stress coping, which focuses on taking constructive action and changing a stressful situation, and is usually associated with problem solving and effective emotion regulation. There was a positive correlation for negative stress coping strategies. The same relationship applied to anxiety [18].

Stress coping strategies and their relationship to the severity of depressive and anxiety symptoms were assessed by Brytek (2007) in a group of patients of Polish and French nationalities suffering from bulimia nervosa. The study included 30 women of Polish nationality and 14 women of French nationality with bulimia nervosa, and a control group of 30 Polish and 17 French randomly-selected female students. The average age of the bulimia patients studied was 21.2 years for the Polish subjects and 22.9 years for the French subjects. The study used the Hospital Anxiety and Depression Scale (HADS) by Zigmond and Snaith, and Carver’s Brief-COPE Inventory. The Polish women with bulimia nervosa had significantly higher levels of depression compared to the healthy female students. The French patients also had higher levels of depression and anxiety compared to the control group. The results of the study revealed the presence of significant differences in stress coping strategies between the experimental and control groups. In the Polish group, these strategies included active coping with stress, planning, behavioral disorganization, withdrawal, positive reframing, humor, denial and use of psychoactive substances. In contrast, in the French group, these strategies included active stress management and behavioral disorganization. The results of the Brief COPE further indicate that the Polish women with bulimia used stress coping strategies based on positive reframing, humor and acceptance less frequently than the French patients. The Polish women surveyed, on the other hand, scored higher in strategies focusing on the use of psychoactive substances [19]. In the results obtained by Brytek, as in the work presented herein, the choice of coping strategies was influenced by depression and anxiety, co-occurring with eating disorders. Depressive symptoms, as well as anxiety symptoms, accompanying another mental illness, can compound the choice of less effective ways to face difficult situations. The patients with anxiety disorders (in our study) also had similar differences compared to the healthy individuals in choosing a coping method in a stressful situation, as did patients with depression. 

Research on coping strategies in young-adult populations seems to confirm their importance in the development of depressive disorders. In their study of 510 Malaysian students, Abdollahi et al. (2018) confirmed that an emotion-focused coping style, avoidance, and judgmental perfectionism may predispose to the development of depression [20]. In an attempt to assess the role of stress coping styles in the development of depression and anxiety, Akhtar et al. (2019) surveyed 122 foreign students of medicine in Germany. Data was collected through an electronic survey using reliable tools, including the Major Depression Inventory (MDI), the Beck Anxiety Inventory (BAI), the COPE Coping Orientation to Problems Experienced Inventory, and a demographic sheet. Reflective coping was found to be a health-promoting coping style, predicting low levels of depression and anxiety among international medical students. Suppressive and reactive coping were considered dysfunctional, as these styles proved to be relatively stronger predictors of high levels of depression and anxiety [21]. 

Strategies for coping with stress have particularly important implications for an individual’s health, including that they can be an important element in preventing the risk of developing or further aggravating abnormal mental states. A study to assess the risk of suicidal behavior of Chinese university students by examining psychological indicators such as hopelessness, happiness orientation, meaning of life, depression, anxiety, stress, and coping styles was conducted in November 2016. A stratified and cluster random sampling method was used to select participants from two large public medical universities in Shandong Province, China [22]. This sample consisted of a large population of 2074 undergraduate students (706 men, 1368 women; mean age = 19.79 ± 1.39 years). The strongest predictors of suicidal behavior were hopelessness, depression, stress and certain coping styles such as self-distraction and self-blame. As a protective factor, the presence of a meaning of life showed potential for suicide prevention. It is noteworthy that the presence of a meaning of life had a positive effect on suicide prevention and acted as a protective factor, which suggests that identifying suicide risk factors, including ways to cope with difficult situations, increases the effectiveness of counseling and suicide prevention programs [22]. In the study described, the Brief COPE Scale (BCS) was used. It is a multidimensional scale [23] that represents 14 subscales, each of which contains two items measuring the strategy one would adopt under a certain type of stressor. They are: active coping, denial, use of informational support, substance use, emotional support, positive reframing, behavioral disengagement, venting, planning, humor, acceptance, religion, self-distraction, and self-blame. 

The purpose of other studies conducted by Ziarko et al. (2011) was to check if the positive relationship between neuroticism and anxiety in rheumatoid arthritis (RA) patients could be moderated by personality variables related to coping with stress, namely coping styles. The authors expected that an avoidant coping style might play a moderating function of the neuroticism-anxiety relationship. Studies showed that the relationship between neuroticism and anxiety is dependent on the coping strategies used, specifically the avoidance-focused style and one of its dimensions, i.e., seeking of social contact [24]. As mentioned earlier, neuroticism, meaning emotional imbalance, is one of the personality traits that predispose to depression, along with traits such as a lack of constructive coping strategies and styles and treating stressors as threats, among others [15].

Man uses various remedial methods not only for different stressors, but also for different aspects of the same stressor. Dealing with stress requires constantly changing efforts to master the demands coming from the environment and those we set for ourselves, often exceeding our capabilities. In the case of depressive-type mood disorders, the symptoms of the disease may be an important factor in the negative assessment of one’s own ability to cope with difficult situations and a greater tendency to perceive events that are a source of stress as a threat that exceeds the ability to cope with it.

In the studies we described, the mean age of patients with depression was significantly higher than that of those with anxiety disorders and that of healthy subjects, which may limit the accurate analysis of the variables in question and thus represent some limitation of the presented research. However, this does not diminish the importance of mental illness in the selection of less favorable coping strategies used, as the correlation of age with the chosen coping strategies for each group and the population as a whole did not significantly project the type of coping methods and styles chosen. In the case of mental illness, the interpretation of the stressful situation and the ability to cope is usually distorted by the patient’s abnormal mental state, regardless of age [3].

An additional limitation of the presented study is the relatively small number of participants in comparison groups and the fact that they were not equally numerous, which encourages the authors to continue the study. The authors sought to provide a new perspective by conducting an analysis of stress coping strategies among patients with depression. Indeed, this topic is less frequently addressed in scientific research than the evaluation of the stress phenomenon itself and its importance in the course of the disease in question. 

## 5. Conclusions

The research results we obtained can be summarized by the following conclusions:

(1) Patients with depression differed from the healthy subjects in a statistically significant way. Statistical analysis showed that people with depression are less likely than healthy subjects to use *Active Coping, Planning, Seeking of Instrumental and Emotional Social Support, Suppression of Competing Activities, and Positive Reinterpretation*. In contrast, they are more likely to use *Denial, Mental Disengagement*, and *Behavioral Disengagement* compared to those not treated for mental disorders.
(2) Apart from one strategy (*Humor*), there was no statistically significant difference between the patients with the first episode of the disease and recurrent depressive disorders in terms of stress coping strategies.
(3) Compared to the group of patients with anxiety disorders, patients with depressive disorders scored significantly differently on stress coping strategies in only two types of actions taken in stressful situations, namely *Seeking of Instrumental Social Support* and *Seeking of Emotional Social Support*.
(4) As the level of severity of depressive symptoms measured by the Hamilton Rating Scale increased, the tendency to choose typical coping strategies to deal with stress did not rise. Increased levels of mood disorder symptoms were statistically significantly associated with only one strategy used during stressful events, i.e., *Behavioral Disengagement.* The greater the severity of depression, the more frequent the use of this strategy.
(5) Among patients with depression, it was observed that the age of the patients and the duration of their illness correlated with an exceedingly small number of stress coping strategies among the fifteen analyzed.
(6) The gender of the subjects had little correlation with the type of stressful coping strategies chosen.

## Figures and Tables

**Table 1:** Characteristics of the study groups.

Data	Depression (N = 118)	Anxiety Disorders (N = 31)	Healthy Subjects (N = 41)
**Gender**	75 F 43 M	18 F 13 M	26 F 15 M
**Age**	47.82 ± 11.88 (min. 18/max. 66)	36.06 ± 11.50 (min. 19/max. 60)	32.95 ± 11.61 (min. 20/max. 61)
**Age of depression vs. anxiety disorders**	Z = 4.53 *p* = 0.000		
**Age of depression vs. healthy subjects**	Z = 5.85 *p* = 0.000		

F—female, M—male; ±—standard deviation; Z—Mann-Whitney U test; *p*—level of statistical significance.

**Table 2:** Difference between patients with depression and healthy subjects in stress coping strategies.

Variable	M (Mean) Depression (118 Individuals)	M (Mean) Healthy (41 Individuals)	Z	*p*
Active Coping	10.71	11.90	−3.44	0.001
Planning	10.34	12.63	−4.57	0.000
Seeking of Instrumental Social Support	9.95	11.98	−3.90	0.000
Seeking of Emotional Social Support	9.28	10.83	−2.62	0.009
Suppression of Competing Activities	9.98	10.98	−2.13	0.033
Turning to Religion	8.74	7.98	0.68	0.496
Positive Reinterpretation	9.43	11.68	−4.80	0.000
Restraint Coping	10.39	9.80	1.51	0.131
Acceptance	9.62	10.37	−1.38	0.166
Focus on and Venting of Emotions	11.64	11.39	0.49	0.624
Denial	7.43	6.05	3.19	0.001
Mental Disengagement	8.60	7.78	2.44	0.015
Behavioral Disengagement	9.36	6.07	6.06	0.000
Substance Use	6.14	5.51	0.53	0.598
Humor	5.82	6.61	−1.70	0.088

Z—Mann-Whitney U test; *p*—level of statistical significance.

**Table 3:** Differences between patients with depression and subjects with anxiety disorders in stress coping strategies.

Variable	M (Mean) Depression	M (Mean) Anxiety Disorders	Z	*p*
Planning	10.71	10.35	0.80	0.427
Seeking of Instrumental Social Support	10.34	10.06	−2.06	0.039
Active Coping	9.95	11.13	0.92	0.359
Seeking of Emotional Social Support	9.28	10.84	−2.45	0.014
Suppression of Competing Activities	9.98	9.84	0.71	0.476
Turning to Religion	8.74	9.03	−0.25	0.799
Positive Reinterpretation	9.43	8.74	1.61	0.108
Restraint Coping	10.39	10.48	−0.39	0.700
Acceptance	9.62	8.84	1.48	0.139
Focus on and Venting of Emotions	11.64	12.03	−0.85	0.397
Denial	7.43	7.16	0.48	0.628
Mental Disengagement	8.60	8.42	0.07	0.940
Behavioral Disengagement	9.36	9.61	−0.40	0.691
Substance Use	6.14	7.58	−1.04	0.297
Humor	5.82	5.29	1.36	0.173

Z—Mann-Whitney U test; *p*—level of statistical significance.

**Table 4:** Duration of illness versus stress coping strategies.

	N Valid	R Spearman	*p*
Illness duration & Active Coping	118	−0.00	0.997
Illness duration & Planning	118	−0.03	0.713
Illness duration & Seeking of Instrumental Social Support	118	−0.07	0.421
Illness duration & Seeking of Emotional Social Support	118	−0.06	0.535
Illness duration & Suppression of Competing Activities	118	−0.12	0.214
Illness duration & Turning to Religion	118	−0.05	0.599
Illness duration & Positive Reinterpretation	118	−0.17	0.064
Illness duration & Restraint Coping	118	−0.08	0.415
Illness duration & Acceptance	118	−0.01	0.943
Illness duration & Focus on and Venting of Emotions	118	−0.05	0.595
Illness duration & Denial	118	−0.21	0.021
Illness duration & Mental Disengagement	118	−0.20	0.032
Illness duration & Behavioral Disengagement	118	0.01	0.892
Illness duration & Substance Use	118	0.02	0.817
Illness duration & Humor	118	−0.24	0.009

N—number of samples; R—Spearman’s rank correlation coefficient; *p*—level of statistical significance.

## Data Availability

Upon request from corresponding author.
